# Transcriptional Responses to Sucrose Mimic the Plant-Associated Life Style of the Plant Growth Promoting Endophyte *Enterobacter* sp. 638

**DOI:** 10.1371/journal.pone.0115455

**Published:** 2015-01-21

**Authors:** Safiyh Taghavi, Xiao Wu, Liming Ouyang, Yian Biao Zhang, Andrea Stadler, Sean McCorkle, Wei Zhu, Sergei Maslov, Daniel van der Lelie

**Affiliations:** 1 Center for Agricultural and Environmental Biotechnology, RTI International, Research Triangle Park, North Carolina, United States of America; 2 Biosciences Department, Brookhaven National Laboratory, Upton, New York, United States of America; 3 Department of Applied Mathematics & Statistics, State University of New York, Stony Brook, New York, 11794–3600, United States of America; 4 State Key Laboratory of Bioreactor Engineering, School of Biotechnology, East China University of Science and Technology, Shanghai, 200237, China; Wageningen University and Research Centre, NETHERLANDS

## Abstract

Growth in sucrose medium was previously found to trigger the expression of functions involved in the plant associated life style of the endophytic bacterium *Enterobacter* sp. 638. Therefore, comparative transcriptome analysis between cultures grown in sucrose or lactate medium was used to gain insights in the expression levels of bacterial functions involved in the endophytic life style of strain 638. Growth on sucrose as a carbon source resulted in major changes in cell physiology, including a shift from a planktonic life style to the formation of bacterial aggregates. This shift was accompanied by a decrease in transcription of genes involved in motility (e.g. flagella biosynthesis) and an increase in the transcription of genes involved in colonization, adhesion and biofilm formation. The transcription levels of functions previously suggested as being involved in endophytic behavior and functions responsible for plant growth promoting properties, including the synthesis of indole-acetic acid, acetoin and 2,3-butanediol, also increased significantly for cultures grown in sucrose medium. Interestingly, despite an abundance of essential nutrients transcription levels of functions related to uptake and processing of nitrogen and iron became increased for cultures grown on sucrose as sole carbon source. Transcriptome data were also used to analyze putative regulatory relationships. In addition to the small RNA *csrABCD* regulon, which seems to play a role in the physiological adaptation and possibly the shift between free-living and plant-associated endophytic life style of *Enterobacter* sp. 638, our results also pointed to the involvement of *rcsAB* in controlling responses by *Enterobacter* sp. 638 to a plant-associated life style. Targeted mutagenesis was used to confirm this role and showed that compared to wild-type *Enterobacter* sp. 638 a Δ*rcsB* mutant was affected in its plant growth promoting ability.

## Introduction

Producing sufficient food for the growing world population is one of the major challenges faced by humanity. In order to achieve this goal, step change yield gains of 10% or more will be required; these go well beyond yield gains of 1 to 2% as currently obtained with plant breeding and plant biotechnology based approaches (http://foodsecuritygroup.com/decreasing-yield/). The last decade has shown an increased commercial interest in microbe-based solutions to improve agricultural productivity. This includes the application of plant-associated microorganisms, both fungi and bacteria, as biofertilizer to improve nutrient availability and uptake, as biopesticide to control plant pathogens, and as bioinsecticide to control a variety of pests. For example, the global market for biopesticide was valued at $1.3 billion in 2011 and is expected to reach $3.2 billion by 2017, growing at a CAGR of 15.8% from 2012 to 2017 (http://www.marketsandmarkets.com/Market-Reports/biopesticides-267.html).

Endophytic bacteria, such as bacterium *Enterobacter* sp. 638 [1], are ubiquitous in most plant species, where they are residing or actively colonizing the living tissue of their host plants without substantively harming them [2]. *Enterobacter* sp. 638 is an endophytic plant growth promoting gamma-proteobacterium that was isolated from the stem of poplar (*Populus trichocarpa* x *deltoides* cv. H11–11), a potentially important biofuel feed stock plant [3]. In addition to colonizing poplar, strain 638 was also found to promote the growth and establishment of tomato, pepper, wheat, corn and rice [4].

The *Enterobacter* sp. 638 genome sequence reveals the presence of a 4,518,712 bp chromosome, a 157,749 bp plasmid (pENT638-1) and a recently discovered 2,372 bp plasmid (pENT638-2; Genbank accession No. JX026951). pENT638-2 contains two genes coding for proteins related to plasmid replication and recombination, which shows close similarity to functions on plasmid pHW126 (Genbank accession No. FN429030) found in *Rahnella* sp. [5]. Genome annotation and comparative genomics [1] allowed the identification of an extended set of genes specific to the plant niche adaptation of this bacterium, including genes that code for putative proteins involved in survival in the rhizosphere (to cope with oxidative stress or uptake of nutrients released by plant roots), root adhesion (pili, adhesion, hemagglutinin, cellulose biosynthesis), colonization/establishment inside the plant (chemotaxis, flagella, cellobiose phosphorylase), plant protection against fungal and bacterial infections (siderophore production and synthesis of the antimicrobial compounds 4-hydroxybenzoate and 2-phenylethanol), and improved plant growth and development through the production of the phytohormones indole acetic acid, acetoin and 2,3-butanediol.

The genetic determinants required for sucrose metabolism (*scrKYAB*) and the synthesis of acetoin and 2,3-butanediol (*budRABC*) were found to cluster on genomic island 29 of the *Enterobacter* sp. 638 chromosome [1]. Metabolite analysis [1] confirmed by quantitative RT-PCR showed that the production of acetoin and 2,3-butanediol was induced by the presence of plant extracts or sucrose as a sole carbon source but not by lactate in the growth medium, pointing to a close interaction between *Enterobacter* sp. 638 and its poplar host, where the availability of sucrose, a major plant sugar, signals the proximity of its plant host to the bacterium, affecting the transcription of functions important for an endophytic plant-growth promoting life style. This observation made us hypothesize that growth of *Enterobacter* sp. 638 in the absence or presence of sucrose would provide a simplified experimental setup to better understand the bacterial functions involved in the endophytic life style of strain 638. Comparative transcriptome analysis was used in order to gain insights in the differential gene expression profiles for *Enterobacter sp.* 638 when grown under these two distinct conditions. The results confirm the roles of many of the functions previously hypothesized as being important for the endophytic life style of *Enterobacter* sp. 638. Furthermore, analysis of regulatory relationships points to the involvement of *rcsAB* in controlling responses by *Enterobacter* sp. 638 to a plant-associated life style, a role that was confirmed by directed mutagenesis.

## Results and Discussion

### Physiological observations

**Figure 1:**
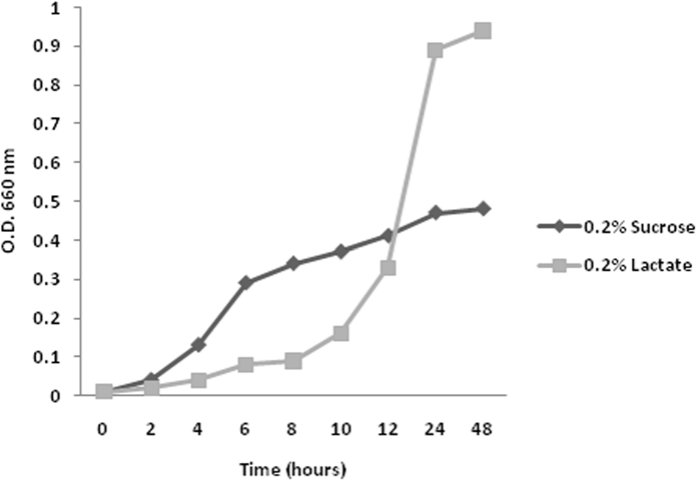
Growth of *Enterobacter sp.* 638 in 284 medium with lactate or sucrose as sole carbon source, respectively. The OD600 was measured in function of time (hours). Lactate and sucrose were present at 0.2% as sole carbon sources.

When growing on Sz-lactate medium, strain 638 showed an exponential growth phase until the culture reaches an OD_660_ of 0.9 after approximately 24 hours (Fig. 1). This growth pattern is in sharp contrast to that observed for strain 638 when grown in Sz-sucrose medium. Cultures growing on sucrose initially grew faster than on lactate, but once reaching an OD_660_ of 0.3 to 0.4 after approximately 6 to 8 hours, they transitioned into the stationary growth phase. After this transition changes in cell behavior were observed: the cells shifted from a planktonic life style to the formation of bacterial aggregates, and cell elongation as was observed by SEM microscopy (First Fig. in S1 File). Furthermore, no increase in cell biomass was observed. It was also found that growth on sucrose let to acidification of the growth medium from 7.0 to 4.4, while cultures growing on lactate had a pH of 8.5 when reaching the stationary phase. Noteworthy, acetoin and 2,3-butanediol synthesis was only observed in medium with sucrose after the cultures entered the stationary growth phase and the pH had dropped, which is consistent with previous reports on optimal conditions for acetoin synthesis from pyruvate [6, 7].

### Differential gene expressions linked to carbon source utilization and energy metabolism

**Figure 2:**
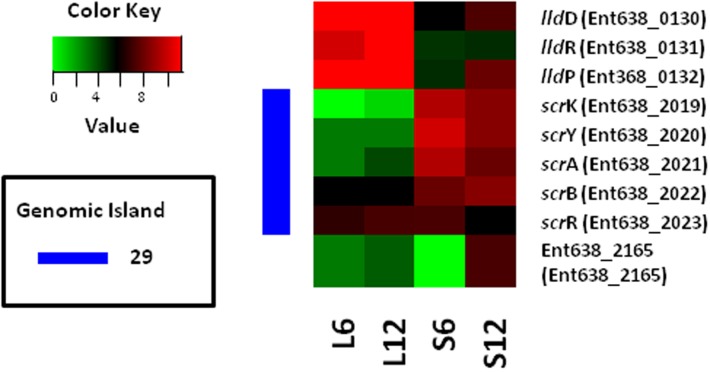
Expression levels of *Enterobacter sp.* 638 genes directly involved in sucrose and lactate metabolisms. Log_2_ of normalized gene expression values (RPKM) are plotted for each condition. L6, L12, S6 and S12 represent the condition lactate and sucrose after 6 and 12 hours growth, respectively. Ent638_2165 is a hypothetical gene that showed homology to *ycjM*, a putative sucrose phosphorylase.

**Sucrose and lactate metabolism.** Genes for carbon source utilization and central metabolism were expressed differently depending on the carbon source present in the medium. After 6 hours, in comparison with growth on lactate, genes of the *scrKYAB* operon for sucrose uptake and metabolism (located on genomic region 29 [1]; Ent638_2019–2022) were 6 to 200 fold induced for cultures growing on sucrose; genes of the *lldPRD* operon (Ent638_0130–0132) for lactate uptake and utilization were 11 to 70 fold induction for cultures growing on lactate (Fig. 2). *Enterobacter* sp. 638 can also metabolize sucrose via phosphorolysis. For cultures growing on sucrose, the expression of a putative sucrose phosphorylase gene (EC 2.4.1.7, Ent638_2165, *ycjM*) was up-regulated 76 fold after 12 hours compared to 6 hours, pointing towards phosphorolysis as the preferred pathway for sucrose metabolism over hydrolysis by the *scrKYAB* operon during the later stages of growth [8].

**Energy metabolism**. Among the various pathways of central energy metabolism in *Enterobacter* sp. 638, the Entner-Doudoroff and the pentose-phosphate pathway show similar levels of gene expression under both growth conditions. Expression of the tricarboxylic acid (TCA) cycle was reduced after 12 hours growth in sucrose medium. In particular, expression of the succinate dehydrogenase gene cluster (Ent638_1221–1229) decreases 7 fold compared with cultures grown in lactate medium. This might reflect the differences in growth phase of the sucrose and lactate cultures, as was shown in Fig. 1: once entering the stationary growth phase, as is the case for growth on sucrose, *Enterobacter sp.* 638 should have lower energy requirement. Furthermore pyruvate, the input for the TCA cycle, is also the precursor in the synthesis of various secondary metabolites, including acetoin, 2,3-butanediol and colanic acid that are important in the symbiotic relationship between *Enterobacter sp.* 638 and its plant host, and whose synthesis levels are significantly increased for cultures growing on sucrose.

### Transcriptional patterns of genes involved in cell signaling and regulation

**Quorum sensing**. In many bacteria, the shift from planktonic growth to biofilm formation is induced by the extracellular concentration of autoinducers (AI-2) [9–11]. In *Enterobacter* sp. 638, the expression level of *luxS*, whose gene product is responsible for AI-2 synthesis, was 1.6 fold induced after 6 h growth on sucrose compared with growth on lactate. After 12 h, *luxS* expression levels had dropped 3.5 fold compared with their levels at 6 h; instead, expression of genes involved in the response to AI-2 (*lsr* operon, Ent638_3531–3538), including *lsrABCD* that code for the AI-2 transporter complex, increased approximately 10 fold between 6 and 12 h growth on sucrose, while expression of *lsrK*, required for phosphorylation of AI-2, increased 4 fold. Elevated levels of phospho-AI-2 will result in de-repression of LsrR synthesis, and subsequently increased expression of *lsrABCD* and uptake of AI-2. Expression levels of *lsrG*, whose protein together with LsrF is necessary for the further processing of phospho-AI-2, decreased 10 fold after 12 hours compared with 6 hours growth on sucrose. Compared with growth in sucrose medium, the expression levels of the *lsr* genes were consistently higher in the presence of lactate at both 6 and 12 hours, especially those for *lsrF* and *lsrG*. These results support the role of quorum sensing in regulating the differences in behavior observed between growth on sucrose and lactate.

**Figure 3:**
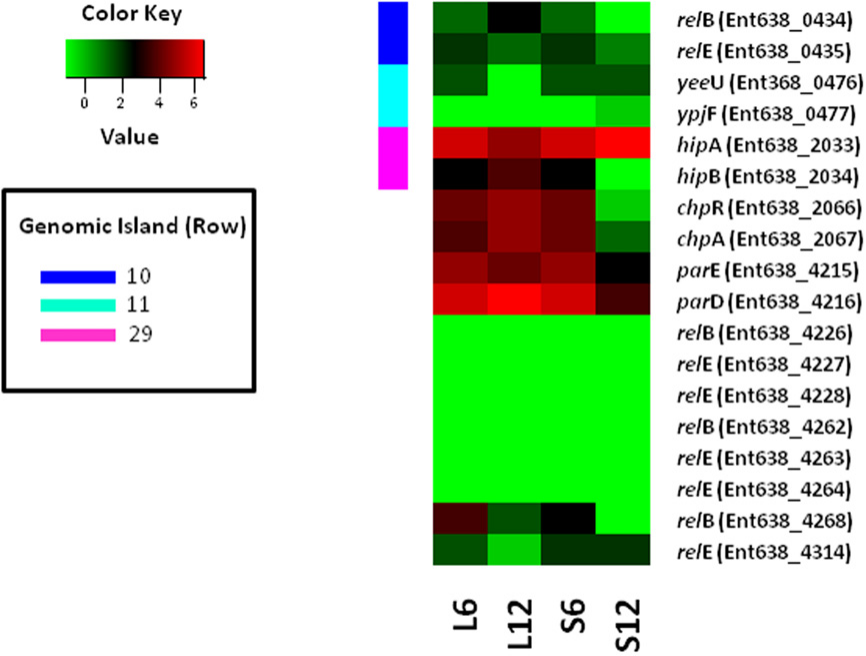
Expression levels of various toxin/anti-toxin systems found in *Enterobacter sp.* 638 in function of carbon source. Log_2_ of normalized gene expression values (RPKM) are plotted for each condition. L6, L12, S6 and S12 represent the condition lactate and sucrose after 6 and 12 hours growth, respectively.

**Toxin/anti-toxin systems.** Toxin/antitoxin (TA) systems are believed to be stress-response elements that help cells survive unfavorable growth conditions [12]. In *E. coli*, AI-2 stimulates biofilm formation and changes its architecture by stimulating flagellar motility via MqsR [13], a regulator which acts as a toxin as part of the *mqsRygiT* toxin-antitoxin system [14]. Although a *mqsR* homolog is absent, the *Enterobacter sp.* 638 genome encodes ten toxin/antitoxin (T/A) systems, four of which are located on the chromosome (*relE/B*, Ent638_0434–35; *yeeV/U*, Ent638_0476–77; *hipA/B*, Ent638_2033–34; and *chpA/R*, Ent638_2066–67), and six on plasmid pENT638-1 (five *relEB-* like and one *parED*) [1]. The expression levels of the genes comprising these T/A systems are either not detectable, similar for both toxin and antitoxin, or show higher levels of anti-toxin expression, with the *hipAB* T/A system [15] being an exception (Fig. 3). In the presence of sucrose after 12 h, expression of *hipA* gene was 2.7 fold induced compared with growth in lactate medium, and was at least 80 fold higher compared to *hipB* expression (which was undetectable). Elevated levels of the HipA toxin, compared to that of the anti-toxin HipB, will result in growth arrest due to a shutdown of macromolecular synthesis, without causing cell death [16]. Thus, the expression pattern of the *hipAB* T/A system might contribute to the sudden shift to the stationary growth phase and the observed cell aggregation and elongation at OD_660_ of 0.4 when *Enterobacter* sp. 638 is grown in sucrose medium. As such the *Enterobacter* sp. 638 *hipAB* T/A system would play a similar role as the *E. coli mqsRygiT* T/A system in controlling cell density. The *hipAB* T/A system is located on genomic island 29, which also harbors the sucrose utilization operon (*scrKYAB*) and the acetoin/2,3-butanediol phytohormone synthesis operon (*budABC*). This observation points to a regulatory mechanism in *Enterobacter sp.* 638 that links sucrose utilization to phytohormone production, *hipAB*-controlled cell growth, and changes in cell morphology.

### Transcriptional patterns of genes involved in motility, biofilm formation and plant colonization

**Motility and chemotaxis**. The *Enterobacter sp.* 638 genome contains multiple flagellar biosynthesis operons as well as determinants involved in chemotaxis, including *flgNMABCDEFGHIJKL* (Ent638_1584–1597), *fliCDSTEFGHIJKLMNOPQR* (Ent638_2522–2541), *flhEAB* (Ent638_2445–2447), *cheZYBR* (Ent638_2452–2455), *tap*, *tar* (Ent638_2456–2457), *cheWAmotBA* (Ent638_2465–2468) and *flhCD* (Ent638_2469–2470), the master regulator of the flagella and motility genes [17]. The expression of these gene clusters was, compared with growth on lactate, reduced for cultures grown in sucrose medium, both after 6 and 12 hours, indicating a reduction in the motility of the cells. This is also consistent with the observed 12-fold decrease on sucrose in *csrA* expression levels (Ent638_3171), whose mRNA-binding protein CsrA [18] acts as an activator of the *flhCD* mRNA, which encodes the master regulator of motility and chemotaxis genes [19].

*Enterobacter sp.* 638 genome contains 6 clusters of type-I pili biosynthesis genes (Ent638_0074–0086, Ent638_0401–0409, Ent638_0987–0994, Ent638_1068–1072, Ent638_2448–2451, Ent638_2458–2462), two clusters of type-IV pili biosynthesis genes (Ent638_0650–0652, and Ent638_3266–3268) as well as a cluster of putative uncharacterized pilus biosynthesis genes (Ent638_3804 and Ent638_3808). Contrary to the expression of flagella and motility genes, the majority of genes associated with the biosynthesis of pili, which are involved in adhesion to surfaces and are among the few factors known to affect endophytic colonization [20], are up-regulated after 12 hours of growth on sucrose. In addition cells grown in sucrose medium were elongated compared with cell grown in lactate medium, presumably due to inhibition of cell division (First Fig. in S1 File). Curli fibers are another factor mediating host cell adhesion and invasion. However, except for the *csgG* gene (2.9-fold induction), no significantly different levels of gene expression were observed for the curli fiber biosynthesis cluster (Ent638_1553–1559).

**Extracellular polysaccharide synthesis.** In *E. coli*, MqsR induces the expression of the transcription factor McbR [21]. McbR inhibits the expression of the periplasmic McbA protein in order to prevent the overproduction of colanic acid; excess colanic acid causes mucoidy, which inhibits biofilm formation [22]. In *Enterobacter sp.* 638, *mcbR* expression decreased for cultures grown in sucrose medium after both 6 and 12 hours. After 6 hours of growth on sucrose, genes of the colanic acid biosynthesis operon (Ent638_2657–2676) became over-expressed 10-fold on average, but after 12 hours gene expression levels were back to those similar as observed for cultures growing on lactate. Previously, it was shown that the increased expression of the colanic acid operon is the genetic response underlying biofilm formation and colonization processes in *E.coli*[23]. In *E.coli* several other genes are induced during biofilm formation, including *ompC* (porin), the *proVWX* operon (high-affinity transport system of glycine betaine), *pepT* (tripeptidase), and *nikA* (nickel high-affinity transport system) [23]. For *Enterobacter* sp. 638, increased expression levels were observed after 12 hours growth in sucrose medium for all these genes, with the exception of the *pepT* gene (Ent638_1640) that showed no significant change in expression levels.

**Acid stress**. Another common trend in the biofilm transcriptome studies is that stress genes are induced [11]. The *asr* gene (Ent638_1915) encoding the acid shock protein was highly induced (40 and 1000 fold) for cells growing on sucrose at both 6 and 12 hours, which is consistent with the observed pH decrease when *Enterobacter sp.* 638 is grown in medium with sucrose as the sole carbon source.

**Stress-related responses.** Plant defenses against microbial infections include the synthesis of reactive oxygen species [24, 25], and *Enterobacter* sp. 638 possesses a variety of systems to defend itself against them [1]. Compared with growth in lactate medium, the expression of the three superoxide dismutase genes *sodA* (Ent638_4063), *sodB*(Ent638_1791) and *sodC*(Ent638_1801) decreased with 50% once the cells entered in the stationary growth phase and shifted to the biofilm stage. Also the expression levels of other functions putatively providing protection against various forms of reactive oxygen, including three catalases, *katE*(Ent638_1712), *katN*(Ent638_3129) and *katG*(Ent638_4032), three hydroperoxide reductases, *ahpC*(Ent638_0872 and Ent638_1145) and *ahpF*(Ent638_1146), two additional hydroperoxide reductases (a putative *ahpC* Ent638_3391 and Ent638_0498 having an AhpD domain), a chloroperoxidase (Ent638_1149), two thiol peroxidases (Ent638_2151 and Ent638_2976) and three glutathione S-transferase (GST) genes (Ent638_0139, Ent638_0268 and Ent638_1329), remained similar or decreased. Only expression of *arcA*, belonging to the *arcAB* (Ent638_0943–0944) locus and presumably required for the export of pytoalexins and the successful endophytic colonization of the host plant [26], went up by 1.5 fold.

**Colonization.** Plant colonization by endophytic bacteria is thought to involve the degradation of pectin/pectate [1], the structural polysaccharide contained between plant cell walls that help the cells bind together. On genomic islands 11 and 29 [1] of the *Enterobacter sp.* 638 chromosome genes are located that putatively encode functions involved in the uptake and degradation of pectate, the demethylated form of pectin. These include a secreted pectate lyase, PelB (Ent638_0482), KdgM (Ent638_0483), involved in the uptake of oligogalacturonides into the periplasm, and a periplasmic pectinase, PelX (Ent638_4134), involved in periplasmic degradation of oligogalacturonide. During the growth in the two different media, these genes were significantly induced (6 to over 20 fold) in the presence of sucrose from 6 to 12 hours, but not in lactate. A 4 to 10 fold increase of expression is also observed for the *uxaABC* genes, which encode enzymes for an alternative pathway for a step in the degradation of pectin: conversion of galacturonate into 2-dehydro-3-deoxy-D-gluconate. Interestingly, the *uxaB* gene (Ent638_2013, genomic island 29) is located closely to the sucrose utilization operon (Ent638_2019–2023), both of which have similar expression patterns.

**Adhesion**. Adhesion forms an important step in the endophytic life style of *Enterobacter* sp. 638. The observed transition from planktonic to biofilm after 12 hours of growth on sucrose might reflect this. The strain 638 chromosome encodes for a variety of cell surface-associated factors that allow adhesion to the host surface, and the transcription levels of most of the genes encoding these functions are significantly increased after 12 hours growth on sucrose. This includes a 10 fold increase of expression of a cluster of five genes encoding for the synthesis of a filamentous hemagglutinin (Ent638_0052–0057) and expression of genes for two putative hemagglutinin proteins (Ent638_0148, 2 fold repressed; Ent638_3119, 4 fold induced). In addition, several genes were found on the chromosome of *Enterobacter* sp. 638 encoding for autotransporter proteins with a pectin lyase/pertactin domain (Ent638_1775, nearly 4 fold induced, Ent638_0318, 7 to 8 fold induced; Ent638_0501, 30 fold induced), or an adhesion domain (Ent638_1867, 4 fold induced; Ent638_3408, 7 fold induced), all of which showed significant increases in gene expression levels after 12 hours growth on sucrose.

**Biofilm formation**. Biofilm formation is an important process in the successful colonization of a plant by its associated microorganisms [1]. The expression patterns of several functions known to be involved in biofilm formation were up-regulated for cells grown in sucrose, including the *cwa* operon for colonic acid biosynthesis and functions involved in pili biosynthesis. The decreased expression of genes involved in motility and increased expression of genes involved in colonization and adhesion suggests that *Enterobacter sp.* 638 becomes less motile under conditions that mimic the association with the plant host. It has been established that motility plays an important role in biofilm development of *E. coli*[27, 28]. Although microarray studies on *E. coli* failed to confirm a significant difference in flagella gene expression during biofilm development [13, 29, 30], other studies have shown that motility genes are repressed in *P. aeruginosa* and *Bacillus subtilis* biofilms [31, 32]. Furthermore, the presence of conjugative plasmids was observed to contribute to increased biofilm formation [33], independent of the expression of motility genes [34]. The transfer functions located on the *Enterobacter sp.* 638 plasmid pENT638-1 (Ent638_4285–4312) were found mostly to be up-regulated after 12 hours growth on sucrose (with average seven-fold change), which might point to a positive effect of pENT638-1 on biofilm formation. Biofilm formation will also provide protection against plant defense systems. Notable is that simultaneously to the observed increases of transcription levels of functions involved in adhesion and biofilm formation, transcription levels of genes that provide protection against various forms of reactive oxygen remained similar or decreased.

### Transcriptional patterns of genes involved in the phytohormone synthesis

**Production of acetoin and 2,3-butanediol**. Consistent with *budABC* gene expression patterns from previous RT-PCR studies [1], cells grown in sucrose medium after 12 hours showed a 1321, 1054 and 283 fold induction of *budABC* genes (Ent638_2026–2028), respectively, compared with cells grown in lactate medium. This result further confirmed the co-regulated expression pattern between the sucrose utilization operon and the acetoin and 2,3-butanediol synthesis operon.

**Production of indole acetic acid (IAA)**. The production of the plant auxin indole acetic acid (IAA) by *Enterobacter sp*. 638 is likely via indole-pyruvate as an intermediate of the tryptophan degradation pathway VII [1, 3]. The key enzyme of this pathway is indole-3-pyruvate carboxylase (IpdC, Ent638_2923), whose expression showed 8-fold induction in the presence of sucrose compared with lactate at 12 hours. No significant differences were observed between cultures grown in sucrose or lactate medium for the expression of the two other enzymes, amino transferase and aldehyde dehydrogenase, of this pathway. However, these two enzymes are considered less important than IpdC, since they are usually found in most bacteria, including those that don’t produce IAA. Furthermore, IpdC was found to be solely responsible for the regulation of this pathway and that the L-tryptophan aminotransferase, which catalyzes the first step in this pathway, operates very close to equilibrium [35, 36].

### Clustering and functional category analysis

To determine the potential involvement of previously unknown functions in the endophytic life style of *Enterobacter* sp. 638, clustering analysis of gene expression patterns was performed using spearman correlation as the distance metric. Five distinctive expression patterns were observed: high level in sucrose but low level in lactate (cluster 1 and 3), low level in sucrose but high level in lactate (cluster 2 and 5), and not much differential gene expressions (cluster 4) (Table 1). For genes in each cluster, functional category analysis (GO analysis) was performed to identify the associated functions with each expression pattern (Table 1). Functions including TCA cycle, motility and chemotaxis were enriched in cluster 2 which is featured by repressed expression levels in sucrose medium. Cluster 4 had no significantly enriched functions. The top representative functional category for Cluster 4 was “unknown functions”. Biosynthesis of surface polysaccharides, colanic acid and pilus are over-representative functions in clusters 1 and 3, which represent high expression level in sucrose medium. Strikingly, functions related to uptake and processing of several nutrients, including N and Fe, also showed up in the clusters 1 and 3 and were up-regulated in the presence of sucrose even with no shortage of these nutrients in the medium. This also included the expression level of *ompC* (Ent638_2795).

**Table 1:** Clustering and analyses of functional categories of genes that were differentially expressed on medium with sucrose or lactate as sole carbon source.

**Bioprocess**	**DR**	**Roles**	**DR**	**Genes per Cluster**
**Cluster 1**				1281
Transport and binding proteins	0.000	The Major Facilitator Super family	0.000	
Prophage functions	0.000	Structural component	0.000	
Plasmid functions	0.000	plasmid transfer	0.000	
Carbohydrates, organic alcohols, and acids	0.011	Replication	0.004	
Scavenge(Catabolism)	0.020	DNA packaging, phage assembly	0.006	
Cations and iron carrying compounds	0.023	Fe aquisition	0.006	
		Pilus	0.008	
**Cluster 2**				1103
Chemotaxis and motility	0.000	Motility	0.000	
Ribosomal proteins	0.000	Ribosome	0.000	
Translation factors	0.000	Translation	0.000	
Surfacestructures	0.001	Flagella	0.000	
ATP-proton motive force inter-conversion	0.002	Cytoplasm	0.000	
TCA cycle	0.046	H+	0.000	
PTS	0.046	Periplasmic space	0.000	
		The H+/Na+-translocating F, V-and A-type ATPase Super family	0.000	
		Tricarboxylic acid cycle	0.024	
		Inhibition/activation of enzymes	0.025	
**Cluster 3**				152
Biosynthesis and degradation of surface polysaccharides and lipopolysaccharides	0.000	Colanicacid(M antigen)	0.000	
Explore	0.000	molybdate	0.033	
Protect	0.000	Methionine	0.033	
Nitrogen metabolism	0.006	Nitrogen metabolism	0.039	
Sugar-nucleotide biosynthesis and conversions	0.026	Dessication	0.039	
Anaerobic	0.046	Glutamate biosynthesis I	0.039	
Siderophores	0.050	Enterochelin (enterobactin)	0.039	
		periplasmic binding component	0.040	
**Cluster 4**				361
-	-	-	-	
				
**Cluster 5**				111
Carbohydrates, organic alcohols, and acids	0.006	4.S.34:citrate/succinate	0.044	

The functional categories of the genes are based on the previously published annotation of the Enterobactersp. 638 genome [1]. Analysis of over-representative functional categories was performed using Goseq 1.0.3. False discovery rate (FDR) values are provided to support the statistical significance of the clustering.

**Nitrogen metabolism**. Although *Enterobacter sp.* 638 is unable to fix nitrogen, it has the genes for the dissimilatory and assimilatory nitrate reduction pathway. Genes encoding the nitrate/nitrite transport and reduction pathways grouped in Cluster 3 and were consistently induced for cells grown in the sucrose medium at both 6 hours and 12 hours. Especially the *nasABnrtCBAnasR* cluster (Ent638_2321–2326), located on genomic island 33, showed an average of 20 fold higher expression level on sucrose than on lactate after 12 hours.

**Iron scavenging**. Siderophores are efficient systems of bacteria to compete for the limited iron, and play an important role in their synergistic interaction and protection of the host plant [37, 38]. In *Enterobacter sp.* 638, the majority of genes associated with enterobactin biosynthesis are located within a large cluster (Ent638_1111–1128), including the siderophore biosynthesis genes (*entFCEBA*), secretion genes (*entS*), and an enterobactin esterase gene (*fes*), as well as two ABC transporter genes for iron uptake (*sitABCD* and *fepCGDB*). The overall expression level of this gene cluster in sucrose medium is higher compared with its expression in the presence of lactate. There are several other transporters involved in the iron uptake that show similar expression patterns, including *fepBGDC*. Most notably, transcription of the *hmu* operon for hemin transport, located on genomic region 25, was over 100 fold induced in the sucrose medium at 12 hours.

**Heavy metal tolerance and metal homeostasis.** The *Enterobacter sp.* 638 genome encodes several putative functions involved in metal(loid) homeostasis and tolerance [1], many of which showed significant changes in expression levels. Up-regulation of gene expression for cells grown in sucrose medium at 12 hours was observed for the P-type ATPAse *copA* (Ent638_0962), the copper efflux operon *cusABCF* (Ent638_1154–1157), the nickel uptake operon *nikABCDER* (Ent638_1834–1839), and P-type efflux ATPase gene *zntA* (Ent638_3873), which is involved in zinc/cadmium/cobalt resistance. The *nikA* gene was shown to be related to the colonization and biofilm formation process [23]. Nickel is also an essential cofactor for urease (*ureABC*, Ent638_3464–3466), which is able to convert urea into ammonia [39]. Consistent with the increased expression of the nickel uptake genes *nikABCDE*, over-expression of *ureA* and *ureC* was observed on sucrose medium at 12 hours.

Overall, functions related to uptake and processing of nutrients, including N and Fe, were up-regulated in the presence of sucrose even with no shortage of these nutrients in the medium. It may suggest for *Enterobacter sp.* 638 to anticipate for limited nutrient resources inside the plant, e.g. resulting from the improved growth and thus demand for essential nutrients by its host [40], as part of the strategy for its commensal life style.

### Analysis of regulatory networks

To visualize the set of transcriptional factors and regulatory interactions potentially responsible for the observed expression patterns 12 hours of growth on sucrose and lactate, the expression differences for S12 —L12 were superimposed onto the regulatory network of the *Enterobacter sp. 638* (see Second Fig. in S1 File). The network in Second Fig. in S1 File shows 835 genes and 1348 regulatory interactions, whose sign (positive, negative, or dual) is in agreement with the observed changes in expression (S12-L12). The five most connected hub regulators, shown in blue, were identified as *Enterobacter* sp. 638 orthologs of the *E. coli* K-12 genes *crp*, *fis*, *ihfB*, *ihfA*, and *fnr* with 175, 87, 71, 71, and 69 connections, respectively. Since these global regulators are broadly controlling cellular processes and responses to environmental changes, such as the availability and type of carbon source, it is hard to hypothesize about their specific role in controlling microbe-host interaction or plant growth promotion.

**The rcsAB two-component regulatory system.** When searching for orthologs of significant regulatory genes from our experiments in the *E. coli* database, it was noted that *gadE*, which encodes the *E. coli* regulator for key functions involved in EPS biosynthesis and the acid response, was lacking in the *Enterobacter sp.* 638 genome. By searching for regulators that share the same targets as GadE, the two-component regulatory system RcsA (Ent638_2542) and RcsB (Ent638_2797) was identified, which is involved in transcriptional control of capsule biosynthesis and cell division. RcsA, a DNA-binding transcriptional activator, showed the highest expression level in sucrose condition after 6 hours. Its targets the *wca* and *yjbEFGH* [41] operons, both of which are involved in EPS synthesis and biofilm formation, showed very similar expression patterns as *rcsA*. The expression of *rcsB*, another positive regulator of capsule synthesis whose activity is controlled by phosphorylation [42], remained at similar expressional levels. The *rcsAB* genes are not co-located on the genome; similar to the organization in *E. coli* the *rcsCD* genes, which are part of the same phosphorylation cascade, were identified adjacent to *rcsB*.

**Figure 4:**
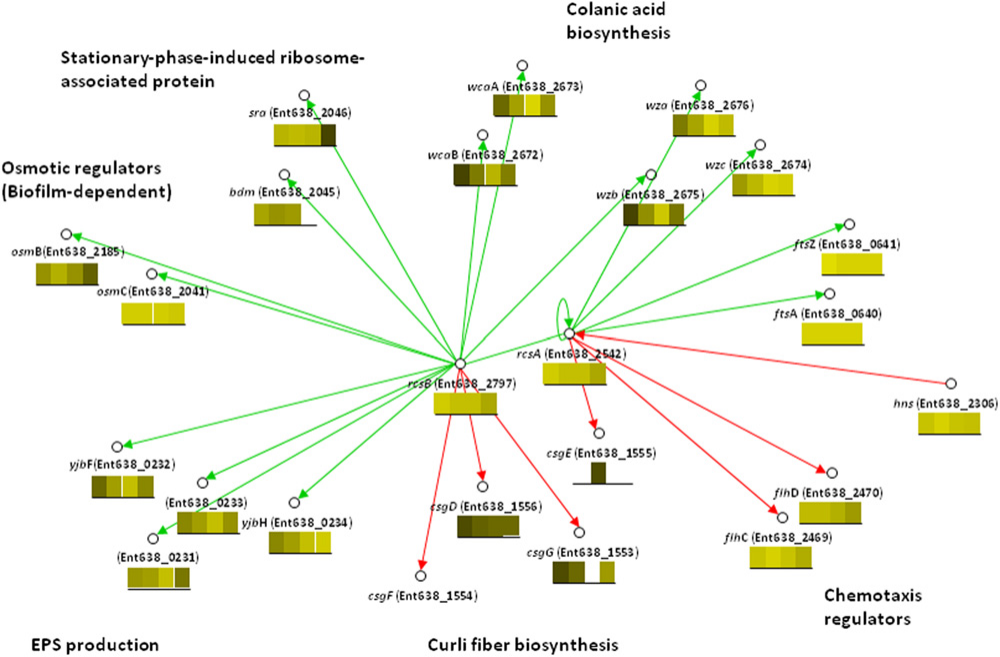
The RcsAB regulatory network in *Enterobacter* sp. 638. In this regulatory network, genes (nodes), gene-gene interactions (edges) and sign of regulation (color of edges: green-activation, red-repression) are projected from regulatory interactions among orthologs in *E. coli* K-12 in RegulonDB v7.5, and were implied from RegulonDB. The blocks under each gene represent its experimentally measured expression levels under the four conditions tested: L6, L12, S6 and S12, corresponding to 6 and 12 hours of growth on lactate and sucrose, respectively. Brighter colors indicate higher expression levels; a blank block indicates that no expression was observed. The figure was generated using VistaClara plugin (1.05) in Cytoscape [63].

Although RcsAB is not necessarily the direct substitute in *Enterobacter sp.* 638 of the GadE regulator, RcsAB is putatively involved in transcriptional control of several processes that are important for plant-microbe interactions. The regulatory connections of RcsAB implied from the *E. coli* regulatory network are shown in Fig. 4. RcsAB appears to activate transcription of genes for capsular polysaccharide and repress genes for flagella synthesis [42]. This is consistent with our observations of activated gene expressions of EPS production and decreased flagella synthesis. Therefore, it is suggested that in *Enterobacter sp.* 638, RcsAB is involved in control of EPS production, flagellar and curli fiber synthesis, functions that constitute an essential as part of the colonization process for bacteria entering host plants.

**Involvement of small RNA regulators.** CsrA together with the small non-coding RNAs CsrB and CsrC (located at positions 3537674–3538029 and 4457944–4458919 respectively on the chromosome), which act as inhibitors of CsrA, coordinate the expression of diverse genes that facilitate adaptation among major physiological phases of growth in various bacteria, including *E. coli* and *Pseudomonas aeruginosa* [19, 43–46]. In addition to regulation of *flhCD*, the best-studied examples of these are the CsrA-repressed *glgCAP*, *cstA*, and *pgaABCD* mRNAs, which in *E. coli* are involved in gluconeogenesis and glycogen biosynthesis and catabolism, peptide transport, and biofilm formation, respectively [18]. The *csrABCD* regulon seems to play a role in the physiological adaptation and possibly the shift between free-living and plant-associated endophytic life style of *Enterobacter* sp. 638: in addition to the observed decrease in *csrA* expression levels, growth on sucrose as compared with 6 hours resulted after 12 hours in a decrease of the *csrB* level, but in an increase of *csrC* transcription. Also the transcription levels of *csrD*, which serves as a specificity factor required for RNase E-mediated decay of CsrB and CsrC [47], was 3.5-fold induced and might account for the observed decrease in CsrB levels. Expression levels of *glgCAP* stayed stable, while no equivalent of the *pgaABCD* operon could be identified in *Enterobacter* sp. 638. Compared with 6 hours, the transcription levels for *cstA* increased 5 fold for 12 hours growth on sucrose.

### Targeted mutagenesis of functions putatively involved in host interaction

Knock-out mutants were constructed for the following genes: *budA* and *budC*, required for acetoin and 2,3-butanediol synthesis, respectively; *hipA*, the toxin of the *hipAB* toxin-antitoxin system; *rcsB;* and *osmC*, which is important in biofilm formation and under positive control of the *rcsAB* regulatory network in *E. coli*. After confirmation of the inactivation of the target gene by PCR and DNA sequencing, the mutants were analyzed for the following properties:

**Acetoin synthesis**. As expected, acetoin synthesis was inactivated for the Δ*budA* strain. The *ΔbudC* strain, which is blocked in the conversion of acetoin into 2,3-butanediol, showed after 48 hours acetoin levels that were 30% elevated as compared with the wild-type strain 638. When compared with strain 638, no effect on the acetoin levels was observed for the other mutants.

**Growth in the presence of sucrose or lactate as sole carbon source.** One of the major problems caused by pathogenic bacteria is uncontrolled growth and blockage of the plants vascular tissue [48]. Therefore, control of cell density during endophytic colonization is very important to avoid pathogenic responses and induction of the plant’s immune response. All strains, including the Δ*hipA* mutant, showed similar growth patterns: when grown in Sz-sucrose medium, all strains entered the stationary growth phase after reaching an OD660 of approximately 0.4, but continued to grow on lactate till reaching an OD660 of 1, indicating that HipA is not the primary function responsible for the observed growth arrest. When using M9 medium, which has a stronger buffering capacity than Schatz medium, all cultures reached a final OD660 of 1, independent of the carbon source (lactate or sucrose), indicating that the decrease in pH for cultures grown in Schatz sucrose is one of the factors triggering the transition into the stationary phase. Other factors such as quorum sensing are hypothesized to contribute to the observed growth pattern. We are also postulating a role for a filamentous hemagglutinin operon (Ent638_0052–0057). Based on sequence homology this function is very similar to the CdiA protein of *E. coli*, which is responsible for contact-dependent inhibition of growth [49, 50].

**Figure 5:**
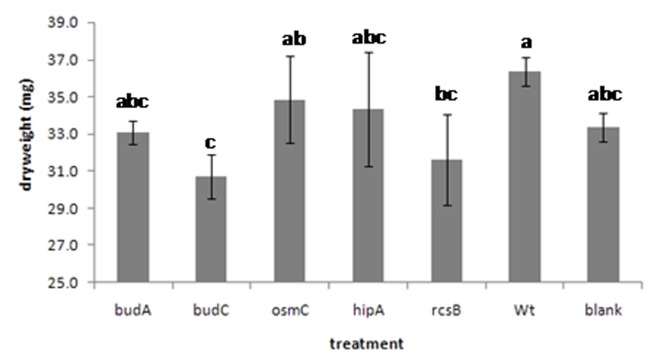
The effects of various mutations on the growth of wheat plants after inoculation with wild-type strain *Enterobacter* sp. 638 and its derivatives. Derivatives containing knock-out mutations for the following genes were examined for their effects on wheat growth: *budA* and *budB*, required for acetoin and 2,3-butanediol synthesis, respectively; *hipA*, the toxin of the *hipAB* toxin-antitoxin system; the *rcsB* regulator; and *osmC*, which is important in biofilm formation and under positive control of the *rcsAB* regulatory network in *E. coli*. The error bars show the standard deviation (SD). Different letters on top of each column represent the significance at the 0.05 level.

**Effects on plant growth and development.** The effect on growth by inoculating wheat seedlings with *Enterobacter* sp. 638 or the mutants was compared with the growth of non-inoculated control plants. Compared with the control, seedlings inoculated with strain 638 showed after 2 weeks the highest increase in biomass. Mutations in the acetoin—2,3-butanediol pathway had a significant effect on the early growth of wheat: seedlings inoculated with the Δ*budC* mutant had the lowest biomass, indicating that the elevated levels of acetoin, as produced by this strain, negatively affected the growth of wheat (Fig. 5). Wheat inoculated with the Δ*budA* mutant, which had lost the ability to produce both acetoin and 2,3-butanediol, showed growth similar to the non-inoculated control plants and a non-significant decrease of 0.27 g in dry weight, indicating that acetoin and 2,3-butanediol synthesis is important for the increased initial growth of wheat as was observed for plants inoculated with *Enterobacter* sp. 638. Inactivation of the *hipA* and *osmC* genes had some effect on the growth of wheat: plants inoculated with these respective mutants grew better than non-inoculated control plants with 1.0 and 1.50 g increase in dry weight, respectively, but less than plants inoculated with the wild type strain 638. On the other hand, plants inoculated with the Δ*rcsB* mutant grew less than non-inoculated control plants, showing the importance of this gene as a regulator in the association of *Enterobacter* sp. 638 and its host plant.

**Figure 6:**
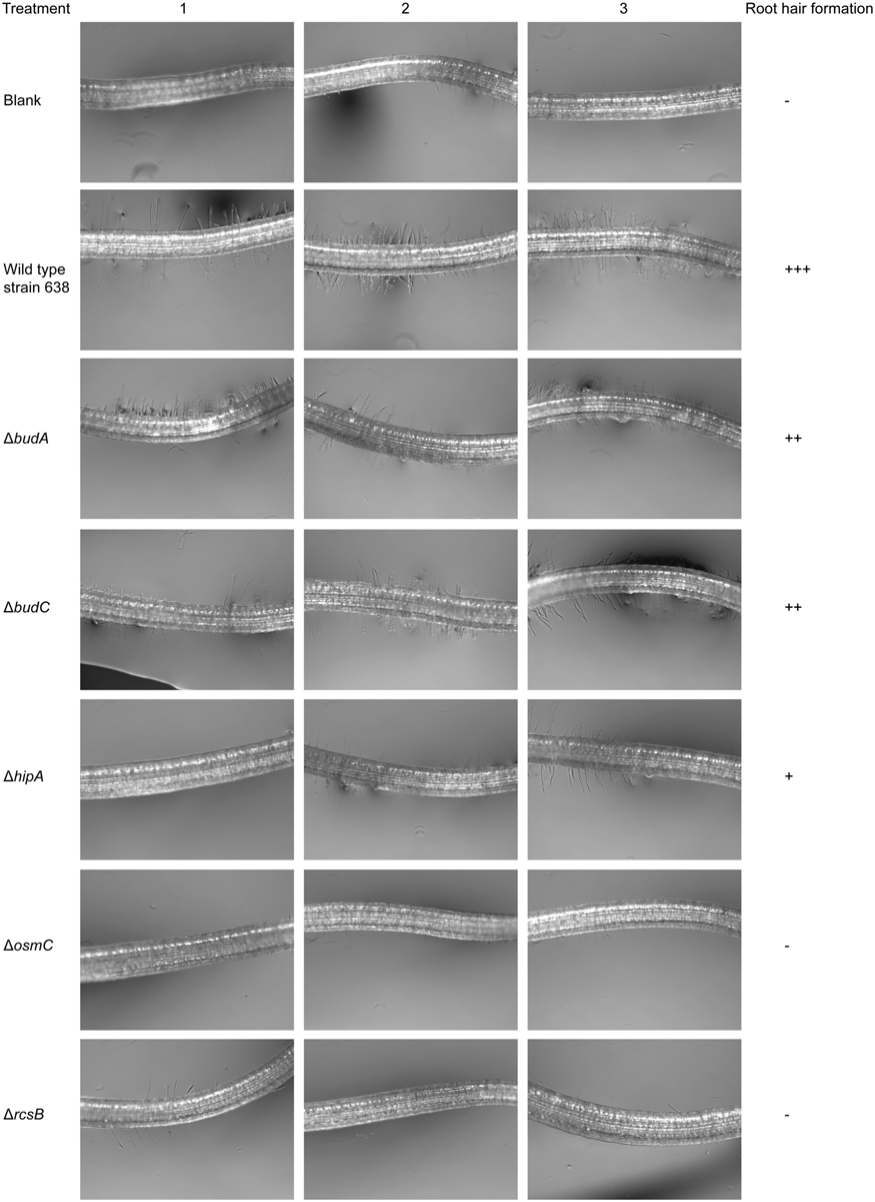
The effects of various mutations on the early development of root hairs by tomato plants after inoculation with wild-type strain *Enterobacter* sp. 638 and its derivatives. The section of the root, 2–3 mm from the root tip, was visualized using light microscopy 48 hours after inoculation. Root hair formation was determined by the average number of root hairs as counted in triplicate within the visual field image of the microscope:-, 0~5 root hairs; +, 6~20 root hairs; ++, 21~40 root hairs; +++, 41~60 root hairs.

The effect of *Enterobacter* sp. 638 and its mutants on early root development was examined using tomato seedlings (Fig. 6). In the case of non-inoculated control plants or plants inoculated with the Δ*osmC* and Δ*rcsB* mutants, no root hair development was observed 48 hours after germination. It was also observed that compared with plants inoculated with the wild-type strain 638, root hair formation was significantly impaired for plants inoculated with the *ΔhipA* mutant, and to a lesser extend for plants inoculated with the *ΔbudA* and *ΔbudC* mutants. It should be noted that after 7 days, all plants had developed root hairs, indicating that the effect of strain 638 is specific during early root development.

The more pronounced effect of the Δ*rcsB* mutation compared with the Δ*osmC* mutation is not surprising, as the inactivation of Δ*rcsB* will affect the expression of several genes, including *osmC*, that have a role in the interaction of 638 with its plant host. However, not all functions involved in the interaction between strain 638 and its plant host are under control of *rcsAB*: levels of acetoin and 2,3-butanediol, important phytohormones whose synthesis is induced by growth on sucrose, were not affected by the Δ*rcsB* mutation.

## Conclusion

Genome analysis combined with gene specific PCR and targeted metabolite analysis had previously pointed to a close interaction between the plant growth promoting endophytic bacterium *Enterobacter* sp. 638 and its plant host, where the availability of sucrose, a major plant sugar, signals the proximity of its plant host to the bacterium, affecting the transcription of functions responsible for the synthesis of plant growth promoting compounds [1]. Therefore, comparative transcriptomics on sucrose, to mimic a plant associated life style, and lactate to represent a plant host-independent life style, was used to further build a catalogue of genes that, based on their expression patterns, are putatively are involved in microbe-host interactions. As such, our data could guide future research on the *in planta* expression of genes involved in various stages of endophytic colonization of plants, both by beneficial microorganisms such as *Enterobacter* sp. 638 as well as pathogenic microorganisms.

Overall, our data show that a tight control of various functions affecting plant growth and development is important for the beneficial interaction of *Enterobacter* sp. 638 on its plant host. For instance, overproduction of acetoin, as observed for the Δ*budC* mutant, had a more negative effect on plant growth than no acetoin and 2,3-butanediol synthesis at all; and for processes underlying stimulated root hair formation, the combined action of several functions is important. This includes the synthesis of acetoin and 2,3-butanediol, as well as other functions, e.g. OsmC that is important for biofilm formation as part of the colonization process. Acetoin is also known to be involved in the induction of systemic resistance [51], thus overexpression of acetoin could negatively impact the endophytic establishment of *Enterobacter* sp. 638 and divert plant resources to defense responses rather than growth. These finding are important, as they point to a beneficial effect of using microorganisms, whose metabolic activities are fine-tuned to the physiological and developmental status of its host plant to promote overall plant growth and development, over the application of individual phytohormones in the form of agrochemicals. It also supports the merits of developing microbial solutions as biofertilizers to improve agricultural yields.

## Materials and Methods

### Experimental design

An overnight culture of *Enterobacter* sp. 638, grown in LB medium at 30°C with 180 rpm agitation, was harvested by centrifugation, washed twice in equal volumes of 10mM MgSO_4_, and suspended in 10mM MgSO_4_ to an OD_660_ of 1. Subsequently, this stock culture was used to inoculate in triplicate 100 ml cultures of *Enterobacter* sp. 638 in Schatz minimal salt medium [52] with sucrose or lactate as the sole carbon source to an OD_660_ of 0.05. Lactate and sucrose were present at 0.2%. Cultures were grown at 30°C, 180 rpm agitation, and 10 ml samples were taken for RNA isolation. Two time points representative for the two stages of growth for Enterobacter sp. 638 on sucrose medium were selected for transcriptome analysis in comparison with cultures growing on lactate as a sole carbon source: the first samples were taken at 6 hours, when both the sucrose and lactate cultures were growing exponentially in the planktonic phase; the second series of samples were taken after 12 hours after culture on sucrose transitioned to the stationary growth phase. Samples are referred to as L6, L12, S6 and S12 and represent the condition lactate (L) and sucrose (S) after 6 and 12 hours growth, respectively.

### RNA preparation, sequencing and read mapping on the genome

Total RNA was isolated using the RNAprotect and RNeasy Midi kit (Qiagen) and processed to remove rRNAs (MICROBExpress kit, Applied Biosystems) and genomic DNA (QuantiTect Qiagen) according to the manufacturers’ instructions. Despite this, the samples still contained significant amounts of 16S rRNA and other rRNAs. The enriched mRNAs were converted into cDNA using the SuperScript Double stranded cDNA Synthesis Kit (Invitrogen).

Libraries for whole transcriptome sequencing on an Illumina GAIIx machine were prepared according to the manufacturer’s instructions using the Illumina cluster kit version 1 (for cluster station) and the SBS version 1 kit (for the sequencing itself). 71 base-pair-long single-ended reads were obtained. The *k*-mer uniqueness for the genome of *Enterobacter* sp. 638 was determined and found to hit 99.7% at k = 30, which encourages read mapping using shorter sequences. Therefore, for higher quality score, 4 nucleotides from the 5′ ends and 31 nucleotides from the 3′ ends were removed of each of the 71nt long reads. The obtained 36 nucleotide reads were mapped to the *Enterobacter sp.* 638 genome using an in-house program which constructs a suffix array of the genome and searches for occurrences of sequences, allowing one mismatch. Locations of unique exact hits (no mismatches) and unique 1-mismatch hits with no corresponding exact hit were collected (see First Table in S1 File for a summary of the mapping). An in-house R script was then used to read these hits and compare them to a table containing the coordinates of the 4395 genes as found in the NCBI annotation of the *Enterobacter* sp. 638 genome sequence. A hit was scored when the sequence of the 36 nucleotide read was fully contained in a gene coding sequence.

### Pre-processing of gene expression measures

In order to derive gene expression level and compare values between conditions, read counts were normalized to adjust for varying lane sequencing depths and potentially other technical effects [53]. Two types of normalizations were preformed: between- and within-library normalizations. Within-library normalization was performed by dividing the summarized counts by the length of the gene [54]. The RPKM (reads per kilobase of exon model per million mapped reads) index was used for comparison between genes [55] as a tool to account for differences in total reads. To compare expression changes of certain gene between samples, gene length bias will cancel out because the underlying sequence used for summarization is the same between samples. But between-sample normalization is still critical for comparing counts from different libraries relative to each other. In this study, we applied the quantile normalization method to the pooled count data. [56]. The results are presented in Third Fig. in S1 File. Under the assumption that the majority of the transcriptome expression level remains similar, while only a small set of genes are responsive to the changes in conditions, the normalization generated similar quantile distribution for every sample to make values from different arrays comparable. In Part A of the Third Fig. in S1 File, before normalization two samples from sucrose 12 hours have overall higher expressions (log(RPKM) is around 8) than other samples (log(RPKM) is around 6), which might be due to variations from amounts of cDNA or systematic handling for each array. In this sense, many genes might be false-positively tested as significant when comparing sucrose 12 hours to other conditions. Thus normalization was applied to make all samples have similar distributions (Part B of the Third Fig. in S1 File). The normalized data were subsequently rounded to produce integer values as genes expression level for further differential expression analysis. A full overview of the transcription data is provided in S2 File.

### Differentially expressed gene analysis

Differentially expressed genes were identified using edgeR package [57], using the exact test that has strong parallels with Fisher’s exact test to test differential expressions and compute exact *p* values. The significance level was controlled by false discovery rate at 0.05 [58]. By normalized gene expressions, we performed four pair-wise comparisons—sucrose verse lactate after 6 hours, sucrose versus lactate after 12 hours and time point 12 hours versus 6 hours for sucrose condition as well as for lactate condition. The differentially expressed genes were determined by biological significance fold changes as well as statistical significance. False discovery rate is controlled for comparing thousands of genes simultaneously. The results are summarized in Second Table in S1 File. The analog MA plots from the transcriptome sequencing study are shown in Fourth Fig. in S1 File for each comparison.

### Clustering and functional category analysis (GO analysis)

In the current experimental design, four biological conditions were tested, and thus clustering analysis was first performed to group genes with similar expression pattern across the four conditions. Hierarchical clustering was carried out with distance as (1 − spearman correlation)/2. Therefore, the distances range from 0 to 1 with smaller values indicating similar expression patterns. Subsequently functional category analysis was performed to identify enriched functions associated with specific expression patterns. GO-seq (package 1.0.3), which was developed specifically for RNA-seq data, was used [59] based on the manually curated functional categories as determined for *Enterobacter* sp. 638.

### Regulatory network analysis

Since the genome of *Enterobacter sp.* 638 is very close related to *Escherichia coli* K12, orthologs from *E. coli* K12 were mapped to *Enterobacter sp.* 638 using the KEGG ortholog database within MATLAB (http://www.genome.jp/kegg/). Subsequently, regulatory relationships were inferred in *Enterobacter sp.* 638. The database RegulonDB v7.5 (http://regulondb.ccg.unam.mx/), which recodes the most comprehensive and updated transcriptional network for *E. coli* K12, was used to find regulatory networks in *Enterobacter sp.* 638 based on *E. coli* orthologs. The resulting regulatory networks were customized and visualized using the Cytoscape tool [60] (see Second Fig. in S1 File).

### Mutant strain construction

Knock-out mutants, using site-specific insertion of a kanamycin gene into the *Enterobacter* sp. 638 chromosome, were constructed for the following genes: *budA* and *budC*, required for acetoin and 2,3-butanediol synthesis, respectively; *hipA*, the toxin of the *hipAB* toxin-antitoxin system; *rcsB;* and *osmC*, which is important in biofilm formation and under positive control of the *rcsAB* regulatory network in *E. coli*. This resulted in Δ*budA*, Δ*budC*, Δ*hipA*, Δ*rcsB* and Δ*osmC* knock-out mutants of *Enterobacter* sp. 638. The DNA fragments used for gene inactivation were prepared by a 3-step PCR procedure [61] with some modifications. In brief, the first step involves amplifying the upstream and downstream regions of the target gene (200–400 bp) and the kanamycin resistance cassette with addition of corresponding restriction sites to each fragment (the primer sequences are provide in the supplemental on line materials). The three fragments are subsequently cut by restriction enzymes and then ligated into a linear DNA cassette with the kanamycin resistance gene flanked by regions homologous to the target gene. Finally, PCR amplification is performed to augment the yield of the product. DNA (400 ng/µl) was used to transform electro-competent *Enterobacter* sp. 638 cells, which were first transformed with the recombination-helping plasmid pKD46-RecA (Nature Technology Corporation). Transformants were selected by plating the electroporated cells on LB-Kanamycin (100 µg/ml) plates and further verified by PCR for the correct insertion using three sets of primers: GeneIF/KanR, Gene IIR/KanF, and Gene IF/Gene IIR as described in the supplemental online materials.

### Acetoin test

To determine acetoin production for *Enterobacter* sp. 638 and its Δ*budA*, Δ*budC*, Δ*hipA*, Δ*rcsB* and Δ*osmC* knock-out mutants, strains were grown in Methyl red-Voges Proskauer (MR-VP) medium without kanamycin for 40 h. Subsequently, 1 ml of culture was mixed with 0.6 ml 5% alpha-naphthol solution. 0.2 ml of a 40% KOH solution was added and mixed for 30 seconds. The development of a red color within 15–30 minutes indicates a positive reaction. Using a calibration curve, the acetoin concentration was subsequently determined by adsorption at 530 nm.

### Plant experiments

Wheat seeds were surface sterilized with 1% active chloride for 10 min. After rinsing 3 times with sterilized distilled water, the seeds were placed between sterile wet paper towels and allowed to germinate for 48 h at 24°C. Bacteria were cultured in LB medium, harvested by centrifugation, washed with equal volume of 10mM MgSO_4_, and then suspended to 1×10^8^ cfu/ml in 50 ml half strength sterile Hoagland’s nutrient solution [62] and transferred to a 200 ml Erlenmeyer. To each Erlenmeyer, 20 germinated seeds were added and the top of the flask was covered with foil. To set up for aeration, a glass pipette fitted with a 0.2 µm membrane filter was inserted into each flask, after which air was gently blown into the solution. After 48 hours inoculation time, 10 seeds with similar development were selected for each treatment and then planted in a single pot filled with sand saturated with half strength Hoagland’s nutrient solution. Three replications were performed for each treatment. The plants were allowed to grow for 10 days in a growth chamber (constant temperature of 22°C, relative humidity 65%, and 14/10 hour light and dark cycle, PAR (photosynthetic active radiation) 165 µmol/m^2^s) and watered when necessary. After harvesting and drying for 72 hours at 60°C, the weights of various plant parts were measured.

Tomato seeds (Roma VF) were used to investigate the effects of bacterial inoculation on early root hair development. Tomato seeds were surface sterilized with 0.6% active chloride for 10 min. Preparation of the inocula and germination condition were the same as described for wheat. After inoculation, microscopy (Olympus IX71 system, 40x magnification) was used to examine the effects of the various inocula on root formation and architecture.

## Supporting Information

S1 File Contains the following files. **First Fig.** Scanning Electron Microscopy (SEM) images of *Enterobacter* sp. 638 grown in Schatz medium with lactate or sucrose as sole carbon source. Cells were grown on Schatz minimal salt medium with lactate (A) or sucrose (B) as sole carbon sources for 6, 24 and 48 hours at 30°C. Images were taken at 5000-fold magnification. **Second Fig.** Differences in gene expression between 12 hours of growth in Schatz sucrose and lactate (S12 —L12) medium superimposed onto the regulatory network of *Enterobacter* sp. 638. The approximation to this network was obtained by projecting the set of direct transcription regulatory interactions between genes in the *E. coli* K12 genome onto their orthologs in the *Enterobacter* sp. 638 genome. Red edges indicate a negative regulatory relationship, while green edges indicate a positive regulatory relationship. **Third Fig.** Histogram of gene expression level log2(RPKM) (A) before normalization; (B) after quantile normalization. RPKM: Reads per Kilobase of gene per Million mapped total reads. **Fourth Fig.** MA plots of four differential gene expression comparisons. Y axis: the log-fold change is plotted against x axis: the log-concentration for each gene. Concentration is defined as the proportion of reads of one gene among total reads in that sample. The genes with log-fold change greater than 1 and controlled FDR less than 0.1 (log value of-1.0) are highlighted in red. A smear of points at the left-most edge of the plot represents genes which have zero counts in one of the conditions. **First Table.** Sequence mapping summary. **Second Table.** Differentially gene expression analysis summary. The analysis was done using the R package edgeR.
(DOCX)

S2 File Full overview of the transcription data of *Enterobacter* sp. 638 grown in Schatz medium with lactate or sucrose as sole carbon source. Cells were grown on Schatz minimal salt medium with lactate (A) or sucrose (B) as sole carbon sources for 6 and 12 hours at 30°C.
(XLS)
