# Supplementary material for: Transcriptional Responses to Sucrose Mimic the Plant-Associated Life Style of the Plant Growth Promoting Endophyte Enterobacter sp. 638
Source: PLoS One. 2015 Jan 21;10(1):e0115455. doi: 10.1371/journal.pone.0115455 (PMC4301647; doi:10.1371/journal.pone.0115455)
Supplement: S1 File — First Fig. Scanning Electron Microscopy (SEM) images of Enterobacter sp. 638 grown in Schatz medium with lactate or sucrose as sole carbon source. Cells were grown on Schatz minimal salt medium with lactate (A) or sucrose (B) as sole carbon sources for 6, 24 and 48 hours at 30°C. Images were taken at 5000-fold magnification. Second Fig. Differences in gene expression between 12 hours of growth in Schatz sucrose and lactate (S12 —L12) medium superimposed onto the regulatory network of Enterobacter sp. 638. The approximation to this network was obtained by projecting the set of direct transcription regulatory interactions between genes in the E. coli K12 genome onto their orthologs in the Enterobacter sp. 638 genome. Red edges indicate a negative regulatory relationship, while green edges indicate a positive regulatory relationship. Third Fig. Histogram of gene expression level log2(RPKM) (A) before normalization; (B) after quantile normalization. RPKM: Reads per Kilobase of gene per Million mapped total reads. Fourth Fig. MA plots of four differential gene expression comparisons. Y axis: the log-fold change is plotted against x axis: the log-concentration for each gene. Concentration is defined as the proportion of reads of one gene among total reads in that sample. The genes with log-fold change greater than 1 and controlled FDR less than 0.1 (log value of-1.0) are highlighted in red. A smear of points at the left-most edge of the plot represents genes which have zero counts in one of the conditions. First Table. Sequence mapping summary. Second Table. Differentially gene expression analysis summary. The analysis was done using the R package edgeR. (DOCX) [file pone.0115455.s001.docx]

**Transcriptional responses to sucrose mimic the plant-associated life style of the plant growth promoting endophyte *Enterobacter* sp. 638**

**Supplemental on-line materials**

Safiyh Taghavi^1,2,5^, Xiao Wu^2,3^, Liming Ouyang^1,4^, Yian Biao Zhang^2ǂ^, Andrea Stadler^2^, Sean McCorkle^2^, Wei Zhu^3^, Sergei Maslov^2^, Daniel van der Lelie^1,2,5,*^

^1^ Center for Agricultural and Environmental Biotechnology, RTI International, Research Triangle Park, NC, USA

^2^ Biosciences Department, Brookhaven National Laboratory, Upton, NY, USA

^3^ Department of Applied Mathematics & Statistics, State University of New York, Stony Brook, NY 11794-3600, USA

^4^State Key Laboratory of Bioreactor Engineering, School of Biotechnology, East China University of Science and Technology, Shanghai 200237, China

^5^Current address: Center of Excellence for Agricultural Biotechnology, FMC Corporation, Research Triangle Park, NC, USA

* Correspondence should be addressed to DvdL: E-mail: daniel.vanderlelie@FMC.com; Phone: +1-919.316.3532

ǂ This manuscript is dedicated to the memory of our friend and colleague Yian Biao Zhang

**Key words**

*Enterobacter* sp. 638; endophyte; transcriptome analysis; plant growth promoting; micro RNA; *csrABCD* regulon; *rcsAB*

**A)**


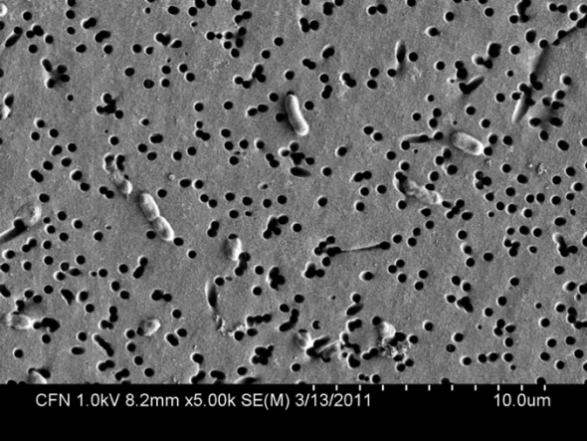

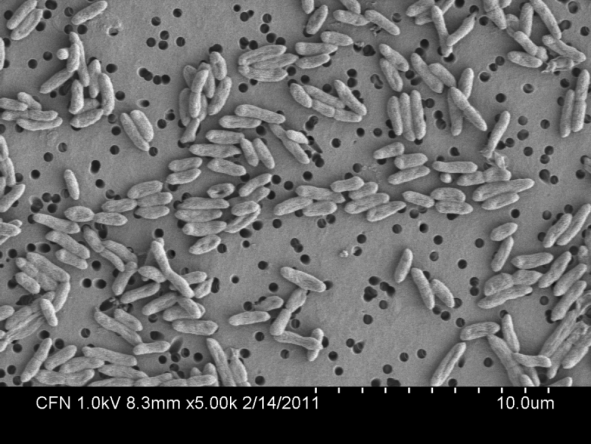

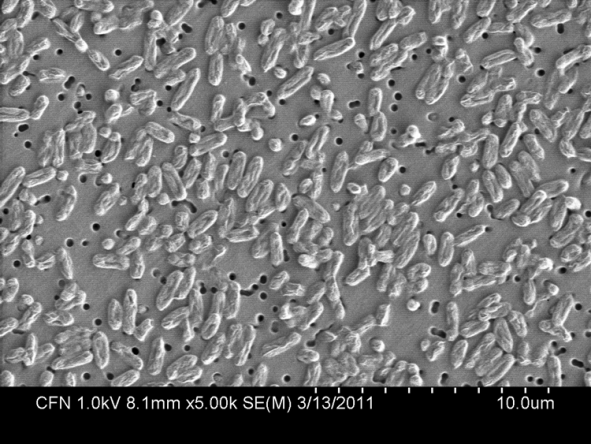


6 hours 24 hours 48 hous

**B)**


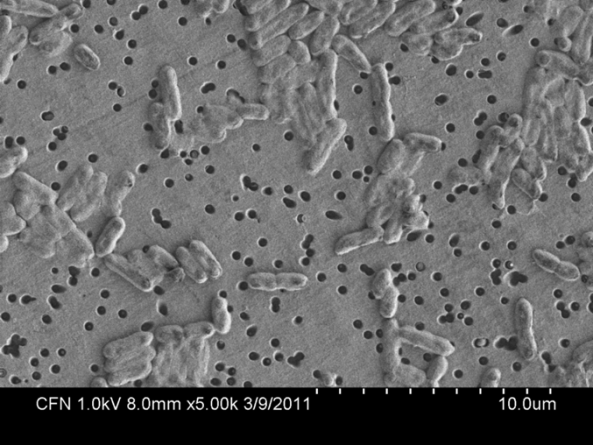

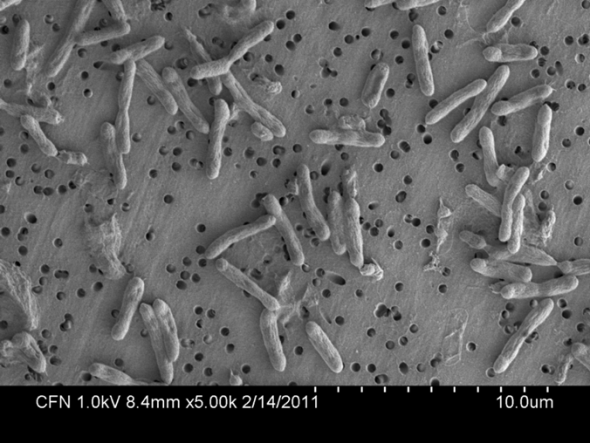

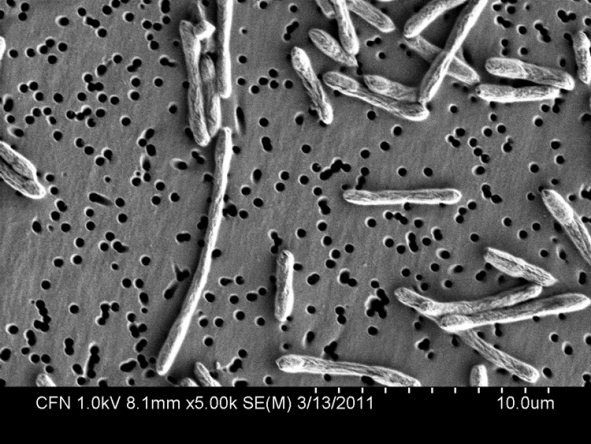


6 hours 24 hour 48 hours

**Figure S1**: **Scanning Electron Microscopy (SEM) images of *Enterobacter*  sp. 638 grown in Schatz medium with lactate or sucrose as sole carbon source.** Cells were grown on Schatz minimal salt medium with lactate (A) or sucrose (B) as sole carbon sources for 6, 24 and 48 hours at 30°C. Images were taken at 5000-fold magnification.

**2. Overview of regulatory network in *Enterobacter sp.* 638**

To identify one or more hub regulators responsible for the observed transcriptional changes, the transcriptional data were superposed on the well-defined regulatory network of *E. coli*, resulting in a regulatory network for *Enterobacter sp.* 638 as shown in **Figure S2**. This figure includes nodes and edges that showed consistent expression (S12 – L12) to the relationships recorded in RegulonDB version 7.5 of *E. coli* K12. A total of 851 nodes were identified.

**Figure S2**: **Differences in gene expression between 12 hours of growth in Schatz sucrose and lactate (S12 – L12) medium superimposed onto the regulatory network of *Enterobacter* sp. 638.** The approximation to this network was obtained by projecting the set of direct transcription regulatory interactions between genes in the *E. coli* K12 genome onto their orthologs in the *Enterobacter* sp. 638 genome. Red edges indicate a negative regulatory relationship, while green edges indicate a positive regulatory relationship.

The set of curated *E. coli* K12 interactions was downloaded from RegulonDB version 7.5. The network in Figure SOM 2 shows 835 genes and 1348 regulatory interactions, whose sign (positive, negative, or dual) is in agreement with the observed changes in expression (S12-L12). The five most connected hub regulators, shown in blue, were identified as *Enterobacter* sp. 638 orthologs of the *E. coli* K-12 genes *crp*, *fis*, *ihfB*, *ihfA*, and *fnr* with 175, 87, 71, 71, and 69 connections, respectively.

**3. mRNA sequencing results**

After applying suffix-array lookup algorithm, we aligned short reads from mRNA sequencing to the reference genome of *Enterobacter sp.* 638 (**Table S1**). Multiple mapped reads are mostly due to seven copies of ribosomal genes. Unmapped reads were examined and found to be patented constructs from Illumina [[1](#_ENREF_1)]. Therefore, uniquely mapped reads are our focus and used for further differential expression analysis. ***A full overview of the transcription data is provided in SOM - Enterobacter sp. 638 gene expression data (Excel file).***

**Table S1:** **Sequence mapping summary.**

|  | Uniquely mapped reads | | Multiple mapped reads | Unmapped reads | Total reads |
| --- | --- | --- | --- | --- | --- |
|  | Perfect Match | One mismatch |  |  |  |
| Lactate 6hr | 4,743,891 | 355,503 | 34,470,352 | 7,974,513 | 47,544,259 |
| Lactate 12hr | 1,447,897 | 68,559 | 30,734,301 | 17,559,946 | 49,810,703 |
| Sucrose 6hr | 2,147,743 | 169,491 | 37,532,613 | 8,670,633 | 48,520,480 |
| Sucrose 12hr | 1,977,053 | 89,197 | 24,152,632 | 2,780,478 | 28,999,360 |

**4. Differential gene expression analysis**

Uniquely mapped reads were then summarized into raw gene expression values by counting the number of reads falling into each defined gene boundary. Raw gene expression values were processed through within normalization RPKM [[2](#_ENREF_2)] and quantile normalization [[3](#_ENREF_3)] (**Figure S3**). Under the assumption that majority of transcriptome should remain similar expression level while only a small set of genes are responsive to conditions, the normalization generated similar quantile distribution for every sample to make values from different arrays comparable. In **Figure S3A**, before normalization two samples from sucrose 12 hours have overall higher expressions (log(RPKM) is around 8) than other samples (log(RPKM) is around 6), which might be due to variations from amounts of cDNA or systematic handling for each array. In this sense, many genes might be false-positively tested as significant when comparing sucrose 12 hours to other conditions. Thus normalization was applied to make all samples have similar distributions (**Figure S3B**).

By normalized gene expressions, we performed four pair-wise comparisons -- sucrose verse lactate after 6 hours, sucrose versus lactate after 12 hours and time point 12 hours versus 6 hours for sucrose condition as well as for lactate condition. The differentially expressed genes were determined by biological significance fold changes as well as statistical significance. False discovery rate is also controlled for comparing thousands of genes simultaneously. The results are summarized in **Table S2**. The analog MA plots from microarray study are shown in **Figure S4** for each comparison. There appears no bias on proportion of significantly differential genes regarding to the expression level of genes.

| (A) Before normalization | (B) After normalization |
| --- | --- |
| 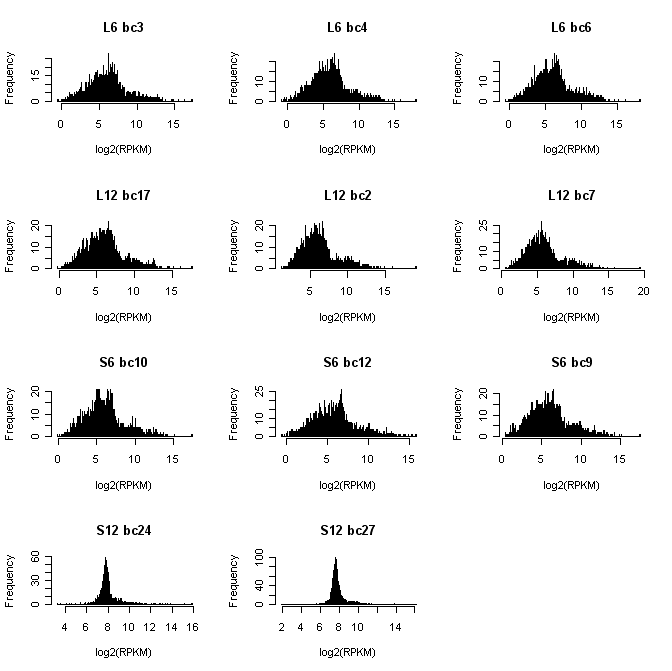 | 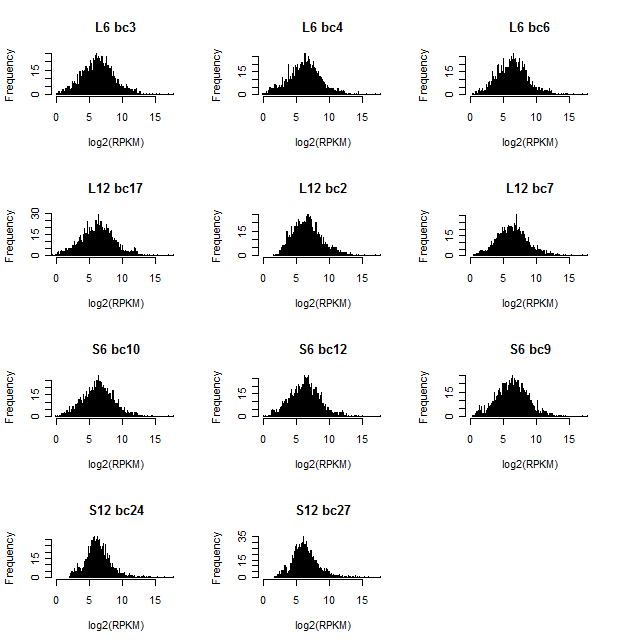 |

**Figure S3**: **Histogram of gene expression level log2(RPKM) (A) before normalization; (B) after quantile normalization.** RPKM : Reads per Kilobase of gene per Million mapped total reads.

**Table S2:** **Differentially gene expression analysis summary.** The analysis was done using the R package edgeR.

| **Condition** | **Controlling FDR < 0.05 (# of genes)** | **FDR < 0.05 & FC >2** | | |
| --- | --- | --- | --- | --- |
|  |  | **# of genes (percentage)** | **Up-regulated** | **Down-regulated** |
| Sucrose-Lactate (6h) | 1528 | 790 (17.4%) | 391 | 399 |
| Sucrose-Lactate (12h) | 2663 | 2392 (52.6%) | 1166 | 1226 |
| 12h – 6h (Lactate) | 385 | 208 (4.6%) | 150 | 58 |
| 12h – 6h (Sucrose) | 3036 | 2423 (53.3%) | 1160 | 1263 |

*FDR: controlled false discovery rate; FC: fold change.

| 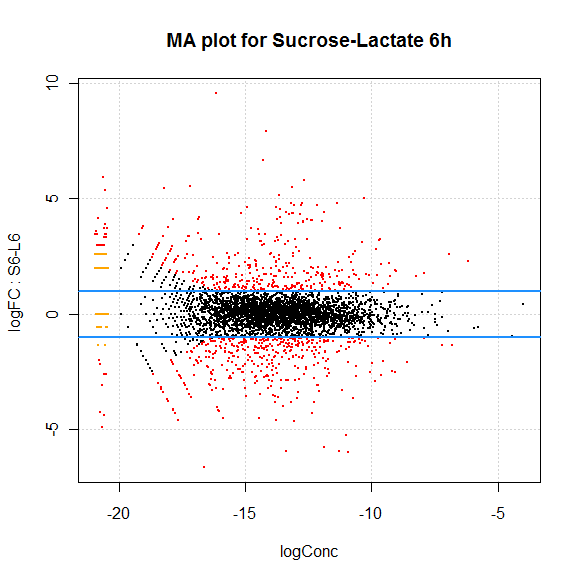 | 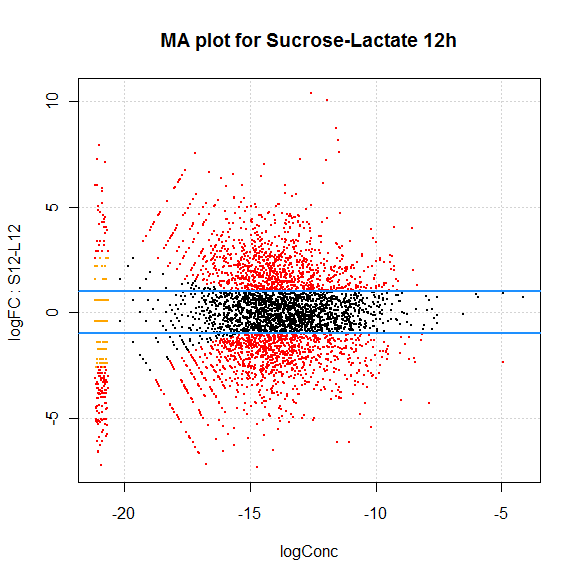 |
| --- | --- |
| 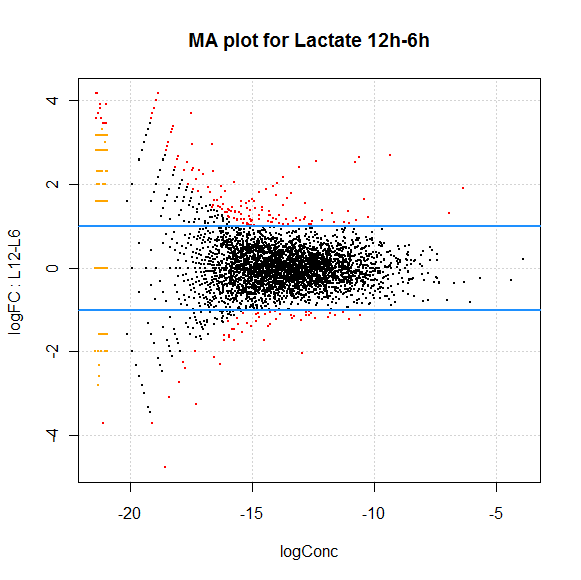 | 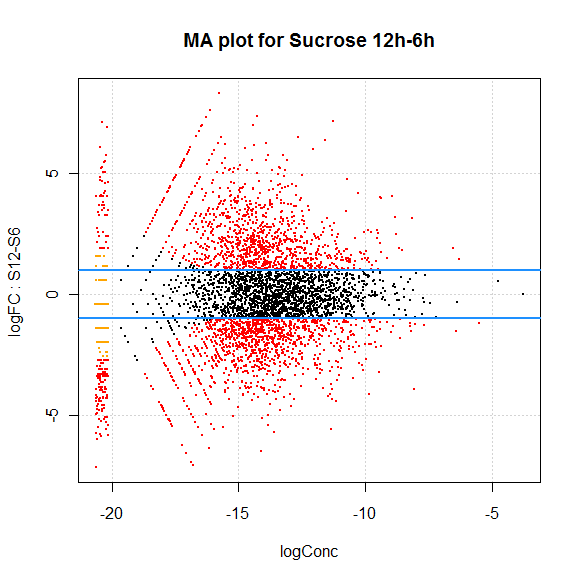 |

**Figure S4:** **MA plots of four differential gene expression comparisons.** Y axis: the log-fold change is plotted against x axis: the log-concentration for each gene. Concentration is defined as the proportion of reads of one gene among total reads in that sample. The genes with log-fold change greater than 1 and controlled FDR less than 0.1 (log value of -1.0) are highlighted in red. A smear of points at the left-most edge of the plot represents genes which have zero counts in one of the conditions.

**5. Primers used for targeted mutagenesis**

1. Kanamycin resistant gene was amplified using the miniTn-5 Km transposon (Epicentre).

KanF: 5’- AAAAAAAAAGCGGCCGCTGTCTCTTATACACATCTCAACC-3’ (NotI)

KanR: 5’- AAAAAAAAATCTAGAGTAATGCTCTGCCAGTGTTACAAC-3’(XbaI)

1. Primers for amplification of target genes

| **Target gene** | **Gene fragment I** | | **Gene fragment II** | |
| --- | --- | --- | --- | --- |
|  | **IF** | **IR (*Not*I)** | **IIF (*Xba*I)** | **IIR** |
| *budA* | GTTGCATTCATCTGCATGCG | AAAAAAAAAGCGGCCGCTCGATCACCTCATGGACTTG | AAAAAAAAATCTAGATAACGTGGCGGGTTATCACG | GTTTTCTACGGAACGGATCG |
| *budC* | GCAAAAAGTTGCTCTCGTAAC | AAAAAAAAAGCGGCCGCGATGTTGTAGACCTTATCGAC | AAAAAAAAATCTAGATAGGGATCACCGTTAATGCC | TTGAATACCATCCCACCGTC |
| *hipA* | GATTTACTGGCAAGCGTTGG | AAAAAAAAAGCGGCCGCTCTGTTCAATTTTGCCGATG | AAAAAAAAATCTAGAAACCAGGATCGCGAAACG | GCCCATTTCAACCATCATCT |
| *osmC* | TCATAAGCACGGTTCAGCAC | AAAAAAAAAGCGGCCGCTGCGCCAATCAACTCTTC | AAAAAAAAATCTAGATACCACTGCGGATGTCTCC | GTGATTTCCGCTTTCAGGAC |
| *rcsB* | ACGACCACCCGATTGTACTG | AAAAAAAAAGCGGCCGCGGGTTATTGTTCATGGTCAG | AAAAAAAAATCTAGATGAAAGCGTTTCTCGACTGC | TCATCGCCGATTTCTTCTGG |

**6. References**

1. Lin B, Wang J, Cheng Y (2008) Recent Patents and Advances in the Next-Generation Sequencing Technologies. Recent Pat Biomed Eng 2008: 60-67.

2. Mortazavi A, Williams BA, McCue K, Schaeffer L, Wold B (2008) Mapping and quantifying mammalian transcriptomes by RNA-Seq. Nat Methods 5: 621-628.

3. Bolstad BM, Irizarry RA, Astrand M, Speed TP (2003) A comparison of normalization methods for high density oligonucleotide array data based on variance and bias. Bioinformatics 19: 185-193.
